# Feasibility of offering nicotine replacement therapy as a relapse prevention treatment in routine smoking cessation services

**DOI:** 10.1186/1472-6963-13-38

**Published:** 2013-02-01

**Authors:** Jessica Turner, Ann McNeill, Tim Coleman, Jo Leonardi Bee, Shade Agboola

**Affiliations:** 1 Division of Primary Care, School of Community Health Sciences, University of Nottingham, University Park, Nottingham, UK; 2 UK Centre for Tobacco Control Studies, Division of Epidemiology and Public Health, University of Nottingham, City Hospital Campus, Nottingham, UK; 3 UK Centre for Tobacco Control Studies and NIHR School for Primary Care Research, Division of Primary Care, University of Nottingham, Medical School, Queen's Medical Centre, Nottingham, UK

**Keywords:** Smoking relapse prevention, Nicotine replacement therapy, Feasibility study, Smoking cessation service

## Abstract

**Background:**

National Health Service stop smoking services (NHS SSS) in the UK offer cost- effective smoking cessation services. Despite high abstinence rates after acute cessation treatment, the majority of clients have relapsed by one year. Several interventions have been identified, from trial data, as effective in preventing relapse to smoking. This study investigated uptake, feasibility and acceptability of offering nicotine replacement therapy (NRT) as a relapse prevention intervention (RPI) in NHS SSS.

**Methods:**

Eligible smokers who had successfully completed acute cessation treatment using NRT at Nottingham City NHS SSS between April 2010 and January 2011 were offered the RPI and the rate of uptake was monitored. Consenting individuals completed a baseline questionnaire, providing demographic and smoking behaviour data. The RPI consisted of using NRT for a further 12 weeks after initial cessation-orientated treatment had ended. At a six-month review, self-reported smoking status was assessed via telephone. Anonymised demographic data on NHS SSS users who did not agree to participate in the study were retrieved from NHS SSS records and used to determine the presence of any socio-demographic differences between individuals who agreed to participate in the study and those who did not. Semi-structured telephone interviews were conducted with a selection of participants; these were audio-recorded, transcribed and analysed to identify participants’ views on the RPI.

**Results:**

Of 493 stop smoking service clients who were assessed, 260 were eligible for and offered the RPI and 115 (44%, CI 38%- 50%) accepted. Individuals who accepted NRT were significantly more likely to be older (p < 0.001) and to pay for their prescriptions (p < 0.001). Quitters who had never worked or were unemployed were significantly less likely to accept the offer of relapse prevention compared to those in routine and manual occupations (55% reduction in odds, p = 0.026).

Interview findings revealed that clients who accepted extended NRT felt the longer duration of pharmacological and psychological support were both valuable in helping them to remain abstinent.

**Conclusion:**

In routine smoking cessation service care, it is feasible to offer clients extended courses of NRT as a RPI. The RPI was acceptable to them as almost half of the eligible clients offered this treatment accepted it.

## Background

Smoking is a cause of significant preventable morbidity and mortality [1]; consequently, reduction in smoking prevalence is a priority for governments and health care systems across the world. The World Health Organisation’s Framework Convention on Tobacco Control (FCTC), the world’s first public health treaty, recommends a comprehensive strategy that participating countries can adopt to reduce tobacco use including advertising bans, the use of taxation and price increases, smoke-free policies, health promotion and mass media tobacco control campaigns, and cessation support. Many countries have smoking cessation services which offer advice and support for smokers to encourage them to quit successfully.

The United Kingdom has implemented a comprehensive tobacco control strategy over recent years [2] including a national network of smoking cessation services, largely provided by the National Health Service (NHS); these services are referred to as the NHS stop smoking services (NHS SSS) (Table 1). NHS SSS commonly provide eight-12 weeks smoking cessation support incorporating pharmacological and psychological therapies to clients who are motivated to quit. Nicotine replacement therapy (NRT), buproprion and varenicline have been proven to be cost- effective with 15% of clients smoke-free at one year [3]. Recent data show 48% of NHS SSS clients have stopped smoking four weeks after setting a quit date [4], however, the majority (85%) have relapsed to smoking by one year, reflecting the addictive nature of cigarette smoking [5]. High relapse rates are common in other similar services offered elsewhere [6]. Effective relapse prevention interventions (RPIs) offered at the end of current treatment could therefore increase long term success rates. Systematic reviews have concluded that RPIs in the form of extended use of NRT, buproprion or varenicline are probably effective when used to prevent relapse amongst smokers who have recently become abstinent [7,8] and economic modelling suggests that this use of extended treatment is likely to be highly cost effective [9]. However, most trial data are from countries without the cessation infrastructure seen in the UK and hence their applicability to routine health care settings such as the NHS is unclear. There is also evidence that smokers trying to stop often use short and incomplete courses of NRT [10,11] and hence identifying the uptake and acceptability of longer courses of treatment is of importance to inform both NHS and similar services internationally. A recent study has identified that, despite there being no evidence to support this view, some providers of NHS SSS are concerned that clients will not accept RPIs that involve using medications for prolonged periods [12]. In this study we therefore investigated the uptake, feasibility and acceptability of offering extended courses of NRT as a relapse prevention intervention to recently abstinent quitters who had been helped to achieve abstinence in a routine health care setting, NHS SSS located across the city of Nottingham.

**Table 1:** Characteristics of stop smoking services

	
**Background**	Established in 1999 in the most disadvantaged areas in England
	Rolled out across the UK from 2000
	Represent a unique national initiative to provide support for smokers motivated to quit [1]
**Service provision**	Service provision framework is based on an evidenced based approach to treating dependent smokers [2]
	Usually involves regular meetings (one to one or in groups) with a trained adviser, using structured withdrawal-oriented behavioural support combined with smoking cessation medications [1]
	Smoking cessation medications are usually offered on the basis of an abstinent-contingent treatment programme involving an initial course of two to four weeks, followed by further prescriptions if the quit attempt is continuing [3]
	If a smokers’ attempt to quit is unsuccessful, advisers can use discretion and professional judgement when considering whether a client is immediately ready to receive support to attempt to stop smoking again [4]
**Training**	Smoking cessation staff come from a wide variety of backgrounds
	They are trained in the provision of treatments to help with stopping smoking
	This training is currently being standardised in England through a newly established National Centre for Smoking Cessation and Training
**Targets**	For the first ten years, targets were set within England for the numbers of smokers attending the services who set a quit date and who quit smoking four weeks after the start of treatment
	Smoking cessation services are currently expected in the course of a year to treat 5% of their local population, in line with best practice recommendations contained within National Institute for Health and Clinical Excellence programme guidance for smoking cessation [3]

## Methods

### Design

Ten NHS SSS clinics were selected from clinics offering specialist intensive smoking cessation support across Nottingham to include a range of sociodemographic catchment areas. The clinics were located within Nottingham City at GP surgeries, Community Centres and Children’s Centres. All clients at these settings who had completed acute cessation treatment eight weeks after their quit dates, and for whom self-reported abstinence was validated with an expired air carbon monoxide (CO) measurement of <8 ppm. were offered study enrolment by their smoking cessation advisor.

### Sample

Eligible clients were those who had used NRT for their acute cessation treatment, were between 18 and 65 years of age and had been regular smokers for 12 months prior to their cessation treatment, and these were offered RPI in the form of extended courses of NRT. Relapse prevention treatment was not offered to those who were pregnant or breastfeeding, individuals with known hypersensitivity to NRT, individuals with unstable cardiovascular disease, unstable cerebrovascular disease including transient ishchaemic attacks, chronic generalised skin disorders or major psychiatric disorders. These individuals were excluded on the basis of information contained within the Nottingham New Leaf Clinical Guidance, (April 2010) which states that clients in these categories are “special precaution groups”. Individuals who used other tobacco products including cigars, pipe tobacco, snuff and chewing tobacco were also excluded. Participants consented to receive extended NRT therapy for up to 12 weeks, to potentially be interviewed about this and to be followed up at six months. Those enrolled completed a baseline questionnaire, which sought their views on using the additional NRT treatment. We also sought permission to conduct follow up at six months for those clients who did not wish to receive extended NRT therapy; these participants were also given a short questionnaire asking about reasons for declining participation.

### Relapse prevention intervention

Individuals who agreed to receive an extended course of NRT were issued with a four week supply of the same NRT product that they had used for acute cessation and they were asked to return in one month for a further four weeks’ supply to be provided by a cessation adviser, contingent on their continued abstinence from smoking (defined as not having smoked more than five cigarettes in the last month, with five or fewer cigarettes being defined as lapses and an expired air carbon monoxide (CO) measurement of <8 ppm). Individuals who reported that they had smoked more than five cigarettes in the month prior to the visit, and with a CO level greater than 8 ppm were not issued additional NRT and were withdrawn from the study.

The following forms of NRT were available for relapse prevention: 24 hour patch, 16 hour patch, 2 mg gum, 4 mg gum, 1 mg lozenge, 2 mg lozenge, 4 mg lozenge, nasal spray and inhalator and 2 mg microtabs. A final month’s NRT was supplied at a third appointment after identical procedures had been followed. Brief counselling support was offered and CO measured at each appointment. Additionally, at each appointment, participants provided information on smoking status, and completed questionnaires asking about the difficulty and acceptability they perceived in using NRT for an extended period NRT, and any side effects experienced.

**Figure 1:**
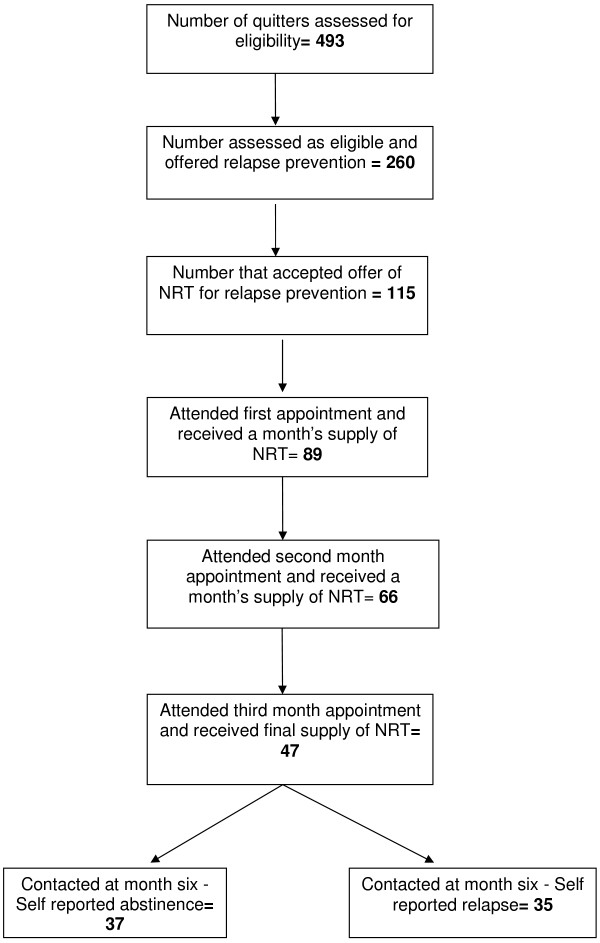
Study flow chart.

At six months, an attempt was made to contact all participants who had agreed to telephone follow up regarding self-reported smoking status with three attempts at calling made; when this was successful, self-reported abstinence (i.e. no cigarettes smoked over the last month) was recorded. The stages involved in the study are described in Figure 1.

### Data access and analysis

Anonymised demographic data on NHS SSS users who did not agree to participate in the study and who did not wish to be contacted at six month follow-up were retrieved from the NHS SSS records and used to determine the presence of any socio-demographic differences between individuals who agreed to participate in the study and those who did not. Data on individuals who refused to participate but agreed to be contacted at six months were also included. These data included age, gender, ethnicity, payment for prescriptions and occupation. Data were retrieved between the months of July 2010 and February 2011.

Data were analysed using STATA statistical software [13]. Summary statistics were performed to estimate the proportion of individuals who accepted the offer of an extended course of NRT (the primary outcome), based on responses to the questionnaire items. Bivariate analyses using chi-squared tests and t-tests were performed to assess any differences in age, gender, ethnicity, occupation and payment for prescriptions between those who accepted the offer of extended treatment and those who did not.

### Qualitative research

Participants who consented to be interviewed were selected purposively to give a range of socio-demographic backgrounds and were then contacted via telephone. Attempts were made to include individuals who were still abstinent, individuals who had relapsed and individuals who had experienced a lapse. Interviews took place over the telephone from May 2010 to April 2011 at between one and four months after they had entered the study. Two different semi-structured interview guides were used; one for clients who had relapsed to smoking and another for those who were abstinent at the time of interview. Guides asked about smoking history, experience of using NRT, the effectiveness of treatment, concerns about taking treatment, consideration of other treatments (specifically bupropion and varenicline), the optimum way of accessing NRT for RPI and circumstances of any lapses to smoking. All who participated in the interviews were offered £10 shopping vouchers.

JT (an author) conducted interviews which were digitally audio-recorded and transcribed verbatim. Transcripts were open coded and a thematic framework was developed, in the context of the study aims, after consideration of themes emerging from the data [14]. Transcripts and the framework were then distributed to all other researchers for review; themes were validated and refined and then re-coded where required. Mapping was then used to look for associations and exceptions. Qualitative data analysis software was not used in the analysis Quotations in the results section are identified by sex, age and occupational status [retired, unemployed/sick, routine and manual, intermediate]/ professional].

### Ethical approval

The study was approved by the Derbyshire Research Ethics Committee, REC reference number 09/H0401/87. The study also received organizational approval from the research and development department of Nottinghamshire County Teaching PCT.

## Results

Of 493 recent quitters seen in the study clinics, 260 were eligible for the offer of relapse prevention treatment and 115 (44%, 95% confidence interval, CI 38%-50%) accepted the offer. Reasons for declining the offer of relapse prevention were given as follows: satisfied with eight weeks’ NRT (n = 66, 46%); inability to attend monthly follow-ups (n = 29, 20%); concern about long-term use of NRT (n = 10, 7%); and ‘other’ (n = 40, 28%). The following explanations were given for those in the ‘other category: “taking champix”, “health problems”, “no longer using NRT”, “special circumstances”, “taking zyban”, and “declined without providing reasons”. The mean age of participants was 43 years, (SD, 11.9 years), most were White British (76%), slightly over half of the participants were female (54%) and around two thirds were exempt from prescription payments (64%).

Table 2 presents participants’ socio-demographic details and Table 3 compares socio-demographic characteristics of those accepting the offer of the RPI with those who did not. Those who accepted NRT were significantly more likely to be older (p < 0.001) and to pay for their NRT prescriptions (p < 0.001). Quitters who had never worked or were unemployed were significantly less likely to accept the offer of relapse prevention compared to those in routine and manual occupations (55% reduction in odds, p = 0.026).

**Table 2:** Sociodemographics of individuals accepting and declining the RPI

**Variable**	**Individuals accepting RPI (n** = **115) (As percentage of variable.)**	**Individuals declining RPI (n** = **145) (As percentage of variable.)**	**Total offered RPI**
			**(n** = **260) (As percentage of variable.)**
**Age**:			
18-25	1 (0.9%)	15 (10.3%)	16 (6.2%)
26-33	11 (9.6%)	23 (15.9%)	34 (13.1%)
34-41	25 (21.7%)	40 (27.6%)	65 (25%)
42-49	30 (26.1%)	27 (18.6%)	57 (21.9%)
50-57	23 (20%)	21 (14.5%)	44 (16.9%)
58-65	25 (21.7%)	19 (13.1%)	44 (16.9%)
**Gender**:			
female	66 (57.4%)	75 (51.7%)	141 (54.2%)
male	49 (42.6%)	70 (48.3%)	119 (45.8%)
**Payment for prescriptions**:			
Paid	41 (35.7%)	23 (15.9%)	64 (24.6%)
Exempt	64 (55.6%)	102 (70.3%)	166 (63.9%)
Unknown	10 (8.7%)	20 (13.8%)	30 (11.5%)
**Ethnicity**:			
White	103 (89.6%)	113 (77.9%)	216 (83%)
Other	11 (9.6%)	15 (10.3%)	26 (10%)
Not recorded	1 (0.8%)	17 (11.8%)	18 (7%)
**Occupation**:			
Full time student	5 (4.3%)	6 (4.1%)	11 (4.2%)
Home carer	9 (7.8%)	13 (9.0%)	22 (8.4%)
Intermediate	6 (5.2%)	10 (6.8%)	16 (6.2%)
Managerial/ professional	3 (2.6%)	5 (3.4%)	8 (3.1%)
Never worked/ unemployed	19 (16.5%)	35 (24.1%)	54 (20.8%)
Retired	13 (11.3%)	4 (2.8%)	17 (6.5%)
Sick/disabled/unable to work	9 (7.8%)	13 (9.0%)	22 (8.5%)
Unable to code	1 (0.9%)	18 (12.4%)	19 (7.3%)
Unknown	3 (2.6%)	2 (1.4%)	5 (1.9%)
Routine and manual	47 (41%)	39 (27%)	86 (33.1%)

**Table 3:** **Comparison of individuals who accepted and declined the offer of relapse prevention treatment** (**univariate analysis**)

**Variable**	**Odds ratio**	**95% CI**	**P**-**values**
**Age**:			0.001
Per year	1.04	1.02-1.06	
**Gender**:			0.363
female	1.26	0.77-2.06	
male	Reference group		
**Payment for prescriptions**:			0.001
Paid	2.84	1.56-5.17	
Exempt	Reference group		
**Ethnicity**:			0.604
White	1.24	0.55-2.83	
Other	Reference group		
**Occupation**:			0.024
Full time student	0.69	0.20-2.44	
Home carer	0.57	0.22-1.49	
Intermediate	0.50	0.17-1.49	
Managerial/ professional	0.50	0.11-2.22	
Never worked/ unemployed	0.45	0.22-0.91	
Retired	2.70	0.81-8.94	
Sick/disabled/unable to work	0.57	0.22-1.49	
Unable to code	0.05	0.01-0.36	
Unknown	1.25	0.20-7.83	
Routine and manual	Reference group		

Of those who agreed to use the extended courses of NRT, 77% (n = 89) attended the one month follow-up and were issued with a second month’s supply of NRT; 57% (n = 66) attended the two month follow-up and were issued with a third month supply of NRT; 41% (n = 47) attended the three month follow-up. At each clinic visit, there was 100% agreement between a participant’s self-reported smoking status and CO measurements, as recorded by the stop smoking advisor (clients are told about CO testing and know to expect it). At the six month telephone follow up, 63% (n = 72) of participants were contacted.

### Ease of usage, acceptability and side effects

Of those followed up in the first month, 98% of individuals reported that they found it “very easy” or “easy” to use the NRT products, this remained at 97% and 98% of individuals finding NRT use “very easy” or “easy” at months two and three respectively. Ninety seven percent of individuals reported that they were “very pleased” or “pleased” with the experience of using NRT at one month; this satisfaction was maintained, with 97% and 96% of those followed up in the second and third month stating they were “pleased” or “very pleased” with the NRT.

A total of 27 (30%) participants reported that they experienced side effects to NRT in the first month, 14 (21%) in the second month and 10 (21%) participants in the third month. The most commonly reported side effects were skin rash from the patches, indigestion and insomnia.

### Abstinence at six month follow-up

Of the 115 participants who took part in the study, 37 (32%, 95% CI 24%-41%) self -reported being abstinent at six month follow-up, 35 (30%, 95% CI 22%-39%) individuals had relapsed and 43 (37%) individuals were lost to follow-up. Only two of the eleven non-participants who consented to be followed up were contactable at six month follow up and both had relapsed to smoking.

### Interview findings

Nineteen interviews were performed. After 16 interviews no new themes were identified and a further three interviews were conducted to confirm data saturation. Participant demographics and relapse/ abstinence status are outlined in Table 4.

**Table 4:** **Socio**-**demographic breakdown of qualitative research interviews**

	**Number of participants**
**Gender**	
Male	5
Female	14
**Age**	
18-25	1
26-33	1
34-41	2
42-49	4
50-57	8
58-65	3
**Smoking status at interview**	
Relapsed	4
Lapse(s)	1
Abstinent	14
**Payment for NRT prescriptions**	
Exempt from prescription charges	11
Pay for prescriptions	8
**Occupation**	
Unemployed/ sick disabled	4
Routine and Manual	7
Professional/ intermediate	3
Retired	5
**Used NHS SSS during previous quit attempts**.	7
**Total number of previous quit attempts**	
None	2
1or 2	7
3 or more	10
**Number of years smoking**	
<10	1
11-20	3
21-30	2
31-40	7
41+	6

In general, the interviewees felt that the RPI was very helpful and they reflected on it positively. The principal themes identified were reasons for continuing quit success, the concept of and reasons for relapse, choice of service, and concerns about prolonged treatment; findings from those themes which relate to the new RPI being evaluated are described in more detail below.

### Reasons for quit success

Participants outlined several factors relating to the extended treatment that they felt had helped them remain abstinent.

The majority of participants perceived that the NHS SSS advisor support was important for practical advice and motivational support, both in the initial cessation period and in the prolonged relapse prevention stage. The use of a carbon monoxide detector was identified as a deterrent to relapse but also as an indicator of physical improvement in the initial cessation stage (Table 5).

NRT was seen as crucial to reducing cravings and physical dependence. Interviewees had several theories as to why prolonged therapy with NRT may have improved their ability to quit and remain stopped. These included the concept that the level of physical addiction was related to the extent of smoking history and that those who had been smoking for longer might need longer to be weaned off smoking. Another concept was that changing ingrained, habitual behaviour is a lengthy process and the longer period of support provided a more appropriate timeframe for this. Other interviewees felt the extended support and treatment was a ‘back-up’ or ‘fail safe’ (Table 5). However, all participants identified that, once a quit attempt had started, NRT alone was not enough to maintain abstinence; self motivation, support from friends, family and formal support services such as the NHS SSS advisors were also important.

**Table 5:** Quotes illustrating ‘Reasons for quit success’

	
**Reasons for quit success**	To start with it was because she was going to do the breathing test… and there was no way I was going to fail that…(F, 63, Retired)
	It’s important to me (speaking to an advisor) because… I know someone is there that I can talk to…(F, 27, Routine / Manual)
	I think for me because I had been a smoker for so long… it was doing it over a long period of time. I think it did me good; I took longer to wean myself down. (F, 63, Retired)
	The government has given you…where they say…we are going to stop just after 12 weeks…it’s too short…too short a time period…because my body has gotten used to say 29…39 years, nearly 40 years of smoking…you can’t do that…stop in 12 weeks… (M, 56, Routine / Manual)
	Yeah, I found erm, I don’t know whether it was just comfort, just to know it was there, you know what I mean? I think if someone had just stuck a plaster on my arm…whether that sounds a bit silly…I just felt I needed it… (Female, 55, Retired)

### The concept of, and reasons for, relapse

Participants who had lapsed or relapsed to smoking often described feelings of guilt and occasionally mentioned feeling physically unwell after smoking. Several participants, regardless of smoking status, identified the theory of ‘social smoking’ or having ‘just one’ as not really being considered smoking. Reasons proffered for previous or current failure in quit attempts were usually related to personal stress. Some individuals recognized that lack of motivation or support from friends, family or partners was also a contributing factor, especially during previous quit attempts conducted on their own (Table 6).

**Table 6:** Quotes illustrating ‘Concept of, and reasons for, relapse’

	
**Concepts and reasons for relapse**	So I went outside…realized what I was doing…and got rid of it…and I felt guilty then for doing it… (F, 52, Routine/Manual)
	The last one was a month ago and before that I was smoking 20 a day. So although I have had one I don’t really consider that as smoking…(F, 39, Routine/ Manual)
	There was one particular incident where I had given up, and then my father-in-law’s step mum became seriously ill and I went to care for her. And in all fairness to get me through that I started smoking again…(F, 50, Sick/ Unemployed)
	I think it didn’t work before because, I think it needed to come from me more. I don’t think I put my heart into it before. (F, 39, Routine/ Manual)

### Choice of service

NHS SSS face to face clinics were the preferred format and provider of support for relapse prevention treatment; this was because they were considered convenient, were free and the service was often considered to be better quality than at alternative providers such as pharmacies and general practices. Some patients mentioned the importance of not having to pay for prescriptions for NRT, and this often determined their response as to where they would like to receive medication from.

Whilst it was recognized that, for some people, face to face appointments at NHS SSS were more difficult to attend, the majority of interviewees felt that these were of added benefit because carbon monoxide readings could be performed and communication was easier. However, there was some enthusiasm for support provided via telephone amongst interviewees too (Table 7).

**Table 7:** Quotes illustrating ‘Choice of service’

	
**Choice of service**	I think it should be free though…for everyone not just people like me on benefits (F, 39, Routine/ Manual)
	For me it was the clinic and that was it. If it wasn’t for the clinic I think I would still be smoking. (F, 60, Routine/ Manual)
	That protected time with a professional therapist. I think it’s very important to have that protected time, even if it’s for only a few minutes, where you know it’s just you and your mentor (F, 56, Intermediate/ Professional)
	I think the (local NHS SSS) is better because… you get to talk to your advisor…if you just go to the pharmacy there’s other people there…you might not get much time because the pharmacy is busy…(F, 21, Unemployed)
	No, I think it has to be face to face… and you have to blow into that carbon monoxide indicator…(F, 49, Intermediate/Professional)
	I don’t mind the telephone thing at all… I finish work at 2 o’clock it saves me from going back out again…(M, 56, Routine/Manual)

### Concerns about prolonged NRT use

No patient stated concerns about continuing NRT for a longer period of time. However, being able to stop using nicotine replacement entirely was seen as desirable as a long-term goal.

## Discussion

### Main findings

To our knowledge this is the first study to attempt to quantify the potential demand for relapse prevention treatment within routine smoking cessation care. We found that 44% of eligible clients who had achieved cessation using NRT and NHS SSS support accepted an offer of extended treatment with NRT; of these 41% continued using NRT for 12 weeks. These findings suggest that there would be a reasonable demand for RPIs (at least among those using NRT for acute cessation) if they were to be routinely offered by NHS SSS. Overall, the qualitative research indicated that participants were generally satisfied with the RPI that they had received, welcoming both the extension of NRT supplies as well as the continuing contact with the NHS SSS advisors and there were few concerns about the extended use of NRT.

### Strengths and limitations

The study was carried out in a selected sample of NHS SSS in one large city and may not therefore be representative of all smoking cessation services in the UK or elsewhere. Nevertheless, we selected services covering a range of socio-demographic areas and this is borne out by the data presented in Table 2. The inclusion and exclusion criteria were based mainly on the stop smoking service supply criteria for NRT but did result in around half of the clients being excluded. Further research is necessary with some of the excluded groups, such as those with psychiatric disease and for extended treatments may be particularly important, given the high rates of heavy dependent smoking in these groups. As such it excluded pregnant women and individuals younger than 18 years and older than 65 years and we were unable to assess the feasibility and acceptability of NRT for relapse prevention in these groups. On the other hand, we have no reason to believe that including individuals younger than 18 years and older than 65 years would have altered the findings in terms of feasibility and acceptability. NRT is used only on the advice of a health professional in pregnancy, and further research is still needed to demonstrate its effectiveness and safety as a relapse prevention intervention in pregnant smokers.

Of those entered into the study, 32% (95% CI 24% to 42%) self-reported being abstinent at six month follow up, but the design of the study was such that it is not possible to determine the efficacy of the RPI treatments provided. This is because no control group (i.e. one not offered RPI) was included and, hence, one cannot be certain about the numbers of extra smokers who remained abstinent at six months because of the RPI. Self-reported smoking status has been shown to correlate well with biochemical measurements. Murray et al. (2002) [15] compared biochemical measures with self-report and found that the size of the differences suggests that self-report bias does not appear to result in “seriously misleading findings in a smoking cessation study”. As the purposes of this feasibility study did not include assessing effectiveness, we only used self-reported smoking abstinence at six months.

The method of sampling for the qualitative study meant that only individuals who could be contacted were interviewed and only a few people who had relapsed to smoking were included. This is a potential source of selection bias and it seems likely that individuals interviewed may have been more motivated to quit and respond more favourably to the RPI. We were therefore unable to explore comprehensively the views of participants who had relapsed to smoking. However, the interviews were carried out with a mix of socio-demographics broadly reflecting those eligible for treatment.

The majority of interviews were conducted during the course of relapse prevention, but a few were conducted after relapse prevention treatment had ended. The interview findings do not suggest that the timing of the interviews had any impact on the perceptions of interviewees regarding relapse prevention.

### Important emergent issues

This study demonstrates that with the support of the NHS SSS staff, a RPI involving NRT can be added to existing treatment protocols, and that around half of eligible smokers are likely to request it. This contrasts with the reported perception of likely low demand for RPIs that has previously been articulated by some NHS SSS staff [12], although these perceptions were largely based on the variable experiences of providing non-evidence based, largely non-pharmacological RPIs in the NHS SSS. The acceptance rate of the RPI offered in this study, in contrast to these negative views suggests that using extended courses of NRT is acceptable to NHS SSS clients.

Clients accepting the RPI in this study appeared to represent a relatively deprived group of smokers; around 40% were unemployed, sick or disabled or carers and a further third were routine or manual workers. This may be because Nottingham City is known to have high levels of deprivation [16]. A prospective cohort study of smoking cessation treatment in primary care found that smokers were more likely to receive smoking cessation treatment if they lived in deprived areas, and this may be due to the likelihood that more affluent smokers buy supplies of NRT over the counter [17]. The main reasons given for declining the offer of relapse prevention treatment were that the clients were satisfied with the eight weeks of NRT they had already been supplied or that they were unable to attend the monthly follow ups. Older clients and clients who paid for their prescriptions were more likely to accept the offer of extended support. The former finding is not surprising as, in previous studies, older smokers have been found to be more motivated and more likely to quit smoking than their younger counterparts [18]. It possibly also suggests that RPIs may be more attractive to those who had been smoking for longer. Even though the sample of recent quitters appeared to represent quite a deprived group, the finding that clients who paid for their prescriptions were more likely to accept the offer of RPI may be because clients who paid for their prescriptions were more motivated to quit as they were willing to invest in the quitting process.

Nearly all those who participated in the study reported that they found it easy to use the NRT products. This finding is not surprising as the literature suggests that NRT products are generally easy to use [19]. The side effects reported were also similar to the established side effect profile of NRT and were all mild and transient [20].

Interviewees recognized that to remain successfully quit, multiple factors are involved including: self motivation, pharmacological help, support informally through family and friends and formally through services such as NHS SSS, factors which have been identified previously [21]. Most of the clients interviewed reported having several failed quit attempts before, had smoked for a long duration and tried various pharmacological and non-pharmacological methods previously. Overall clients were satisfied with the RPI they received; they identified that prolonged nicotine replacement and behavioural support were both key to their remaining quit.

## Conclusion

It is feasible to offer a relapse prevention intervention in the form of an extended course of NRT to smokers through an NHS SSS and nearly half of those offered such a treatment are likely to accept it, with 41% using extended NRT for up to three months. This study also shows that abstinent quitters find extended courses of NRT acceptable and easy to use and they are positive about receiving extended NRT as well as extended behavioural support through NHS SSS. Future research should investigate the effectiveness of NRT used as a relapse prevention aid in routine healthcare settings.

## Competing interests

TC declares that, in the last 5 years, he has been paid for consultancy work by Pierre Fabre Laboratories; manufacturers of nicotine replacement therapy. This manuscript has not been discussed with any third parties. None of the other authors declare any competing interests.

## Authors’ contributions

SA, AM, TC & J L-B designed this study; SA and JT conducted the data collection and analysis, and drafted the initial manuscript. All authors were involved in revising, reading and approving the final manuscript.

## Pre-publication history

The pre-publication history for this paper can be accessed here:

http://www.biomedcentral.com/1472-6963/13/38/prepub
